# Does right hemisphere compensate for the left in school-age children with large left middle fossa arachnoid cysts?

**DOI:** 10.1186/s12887-023-04148-1

**Published:** 2023-11-03

**Authors:** Wenjian Zheng, Xueyi Guan, Zheng Lu, Xianchang Zhang, Huina Zhai, Guodong Huang, Jian Gong

**Affiliations:** 1 Department of Pediatric Neurosurgery, Beijing Tiantan Hospital, Capital Medical University, Beijing, 100070 PR China; 2 Department of Neurosurgery, Shenzhen Second People’s Hospital, the First Affiliated Hospital of Shenzhen University Health Science Center, Shenzhen, 518035 Guangdong P.R. China; 3 MR Collaboration, Siemens Healthineers Ltd, Beijing, 100020 PR China; 4 Beijing RIMAG Medical Imaging Center, Beijing, 100029 PR China

**Keywords:** Arachnoid cyst, Cognitive impairment, Functional connectivity, School-age children

## Abstract

**Background:**

To assess the cognitive function changes and brain network neuroplasticity in school-age children having large (diameter > 5 cm) left middle fossa arachnoid cyst (MFACs).

**Methods:**

Eleven patients and 22 normal controls (NC) between 6 and 14 years of age were included. The CNS Vital Signs (CNS VS) were administered for cognitive assessment. The differences of cognitive data and functional connectivity (FC) in resting-state functional magnetic resonance imaging (rs-fMRI) were compared between the patient group and the NC group. The correlations between the altered FC and cognitive data in the patient group were assessed.

**Results:**

Patient group had significantly poorer attention (including Complex Attention, Sustained Attention, Simple Attention, Cognitive Flexibility, and Executive Function) and memory function (Visual Memory and Working Memory) than the NC group (uncorrected *p*-value, *p-unc* < 0.05). Whole-brain local correlation (LCOR) analysis showed an extensively lower LCOR in the patient group (voxel threshold *p-unc* < 0.001, cluster-size threshold of false discovery rate adjusted *p* (*p-FDR*) < 0.001). Functional connectivity (FC) analysis showed that bilateral frontal and temporal lobes connectivity in the patient group was significantly lower than the NC group (*p-FDR* < 0.05). Seed-based FC analysis indicated that there was altered FC between the right temporal lobe and the left temporal-parietal/temporal-occipital area (*p-FDR* < 0.05). In the patient group, most of the altered FC had a negative correlation to the cognitive score, while the FC in the right temporal lobe-left temporal-occipital area positively correlated to Verbal/Visual Memory (*r* = 0.41–0.60, *p-FDR* < 0.05). In correlation analysis between clinical data and cognitive score, the only significant result was a low correlation between cyst size and Reaction Time (-0.30–-0.36, *P-FDR* < 0.05).

**Conclusions:**

School-aged children with large left MFAC showed significantly lower cognitive performance primarily in attention and memory domains. Distinct from neuroplasticity in a unilateral brain lesion, compensation in the healthy hemisphere in MFAC patients was sparse.

**Supplementary Information:**

The online version contains supplementary material available at 10.1186/s12887-023-04148-1.

## Introduction

Primary arachnoid cysts (ACs) are benign cysts that result from abnormal splitting or duplication of the arachnoid membrane during development [1]. The prevalence in children is between 0.3% and 2.6% [2, 3]. One of the most commonly observed AC is the middle fossa AC (MFAC), comprising 47–65% of all types of ACs [3, 4]. Galassi et al. classified MFAC into three types: type I (small), type II (medium), and type III (large) [5].

The identification of most MFACs occurs incidentally [6]. The primary concern is how and to what extent the cyst affects cognitive function. There were several cognitive studies for children having MFAC [7–10]. The converging findings showed that MFAC patients had various cognitive disabilities, such as attention, reasoning, and language impairments, in different neuropsychological assessments. However, the MFAC included in these studies were heterogeneous (different ages, Galassi type, and hemispheres).

Brain can functionally and physically reorganize its structure in response to pathological changes. Grafman described this property as neuroplasticity [11]. It has been well delineated that functional disability caused by tumor or trauma in one hemisphere can be partially compensated by the opposite side [12]. One intriguing question is: for a patient with large MFAC in one hemisphere, will similar neuroplasticity occur on the opposite side?

In this study, we retrospectively reviewed 11 school-age children with large left MFACs (diameter > 5 cm, type III). Their cognitive performance and functional connectivity (FC) in resting-state functional magnetic resonance imaging (rs-fMRI) were compared to 22 healthy children. Neuroplasticity was determined by the correlation analysis between cognitive and image data.

## Materials and methods

### Subjects’ selection

A retrospective survey was conducted on 11 children who received a diagnosis of left MFAC from the department of Pediatric Neurosurgery, Beijing Tiantan Hospital from August 2019 to January 2022. Demographic information was recorded, and cognitive assessments and rs-fMRI were conducted. Patients’ skulls were measured, and parameters including biparietal diameter (BPD) and occipitofrontal diameter (OFD) were recorded. Inclusion criteria for the study were (1) patients between 6 and 14 years old, who were (2) native Mandarin Chinese speakers, for whom (3) the largest diameter of the MFAC was at least 5 cm, and (4) whose parents provided informed consent for cognitive and image evaluations on their children.

A total of 22 healthy children were recruited as normal controls (NCs). We recruited participants from Beijing. The inclusion criteria were: (1) participants between 6 and 14 years old, (2) no history of previous cerebral or systemic diseases.

The study was reviewed and approved by Beijing Tiantan Hospital Institutional Review Board (KY2021-100-02). All methods were carried out in accordance with relevant guidelines and regulations (Declaration of Helsinki). The subjects’ guardians provided written informed consent to participate in this study.

### Cognitive measure

Patients’ cognitive functions were assessed with the Chinese-language version of the Wechsler Intelligence Scale for Children, Fourth Edition (WISC-IV) and CNS Vital Signs (CNS VS). The interval between the assessment of two batteries was no less than 60 min and no more than two days. The NC’s cognitive function was assessed using the CNS VS. All tests were conducted under the instruction of the authors (Xueyi GUAN and Huina ZHAI).

The Chinese-language version of the WISC-IV had normative samples that consisted of 1,100 Chinese children between the ages of 6 and 16 years [13]. The WISC-IV includes four primary indexes, namely Perceptual Reasoning Index (PRI), Verbal Comprehension Index (VCI), Working Memory Index (WMI), and Processing Speed Index (PSI). General Ability Index is a comprehensive score of the VCI and PRI. Full-Scale Intelligence Quotient (FSIQ) is calculated as the sum of the four primary indexes. The WISC-IV standard scores have a mean of 100 and a standard deviation of 15.

The CNS VS provides age-adjusted standard scores for 15 domains [14]. These 15 domains include Composite Memory, Verbal Memory, Visual Memory, Psychomotor Speed, Reaction Time, Complex Attention, Cognitive Flexibility, Processing Speed, and Executive Function, Social Acuity, Reasoning, Working Memory, and Sustained Attention, Simple Attention, and Motor Speed. The Neurocognition Index (NCI) derived from the 15 domain scores represent the overall neurocognitive status of the patient. The standard score of each domain and subtests has a mean of 100 and a standard deviation of 15.

### Cognitive data analysis

Patients’ cognitive performances in the WISC-IV were compared to the normative level (100 ± 15) provided by the scale. While the CNS VS does not provide a standard score for Chinese subjects, patients’ cognitive performances in the CNS VS are compared to the NC group. The standard scores of each domain and index were analyzed with SPSS 17.0 (Chicago, IL, USA). Statistical analyses of means between groups were carried out with independent samples t-test. An uncorrected *p*-value (*p-unc*) < 0.05 was considered significant.

### Rs-fMRI acquisition

The patients’ rs-fMRI was acquired on a 3T scanner (MAGNETOM Prisma, Siemens Healthcare, Erlangen, Germany) with a 20-channel head/neck coil in Beijing Tiantan hospital. The NCs underwent rs-fMRI using a scanner with the same model and an identical protocol, in the RIMAG image center, Beijing. The imaging protocol included a T1 weighted structure imaging with magnetization prepared rapid acquisition gradient echo (MPRAGE) sequence. Resting-state sequences were acquired with an echo-planar imaging (EPI) sequence. The scan parameters for EPI sequence with simultaneous multisclie (SMS) accelation technique were TR = 2000 ms, TE = 35 msec, slices = 69, SMS = 3, % FOV = 100%, voxel size = 2.2 mm isotropic, volumes = 240. The patients were instructed to remain seated with their eyes closed. No sedation was applied during the examination.

The data were preprocessed and analyzed using the CONN toolbox 20b [15] running on MATLAB R2016b version 9.1.0 (MathWorks, Inc., Natick, MA, USA). First, the slice-timing correction was performed. The head motion, global brain signal, white matter signal and cerebrospinal fluid signal were regressed out from the time course of rs-fMRI. The images were then normalized to a Montreal Neurological Institute (MNI) template, and the normalized images were resliced with a target resolution of 2 mm. The normalized fMRI images were then smoothed (8 mm full width half maximum Gaussian kernel). The motion outlier threshold of the artifact detection tool was set at 95th percentiles of normative samples with a motion threshold of 0.9 mm. ART-based identification was applied, and acquisitions with displacement above the threshold were removed [16]. The brain masking process was applied for voxel-level analysis. To eliminate physiological high-frequency cardiac and respiratory noise low-frequency drift, a band-pass filter from 0.01 to 0.1 Hz was used.

Regions of interests (ROIs) were defined using the default atlas in the CONN toolbox atlas. The atlas has a total of 164 ROIs. Images in which movement in any direction exceeded either 3 mm translation or 3° rotation were excluded from the study. All data are resliced to 3 mm × 3 mm × 3 mm voxels and smoothed using a Gaussian kernel with a full-width at a half-maximum of 6 mm. Data were filtered using both a band-pass filter [0.008–0.09 Hz] and linear detrending.

### Rs-fMRI analysis

Seed-based FC maps were generated for each participant. The FC difference between the patient group and the NC group was calculated by independent sample t-test. The cluster-level inference was set based on random field theory, and correction for multiple comparisons across the brain was conducted by controlling the voxel *p-unc* < 0.001 and false discovery rate adjusted *p* (*p-FDR*) < 0.05 levels using CONN’s implementation of the Benjamini-Hochberg algorithm [17].

Correlations between the Z-transformed score of FC and cognitive data were calculated. Then the Spearman correlation coefficient (*r*) was used as the strength of the monotonical association. An *r* value in the range 0.7–1.0, 0.5–0.7, 0.3–0.5, or < 0.3 in the correlation analysis was regarded as “high correlation”, “moderate correlation”, “low correlation”, or “negligible/no correlation”, respectively [18]. A *p-FDR* < 0.05 was considered statistically significant. The results were displayed using “ggplot” in R.

Local correlation (LCOR) was defined as the average of correlation coefficients between each individual voxel and a region of neighboring voxels [19]. A Kernal size of 25 mm was applied. The significance level was set at the individual voxel *p-unc* < 0.001, and a cluster-size threshold of *p-FDR* < 0.05.

## Results

### Demographic and clinical characteristics

A series of 11 consecutive patients were eligible for the study. There were nine males and two females. Ages ranged from 6 to 14 years old. All patients received the WISC-IV and CNS VS assessment. Patients’ demographic information and clinical characteristics appeared in Table 1. For continuous variables, values were expressed as mean ± standard deviation. For dichotomous variables, values were presented as numbers and percent of cases. The age (10.8 ± 3.1 vs. 10.8 ± 3.0 years old, *t* = 1.399, *p-unc* = 0.172) and gender (male/female, 9/2 vs. 14/8, Fisher’s exact test *p-unc* = 0.430) were compatible between the patient group and the NC group. There was no significant difference in handedness, education level and parental education level between two groups.

**Table 1:** Middle fossa arachnoid cyst patients’ demographics and clinical characteristics

Variable	
**M/F (No.)**	9/2
**Mean age ± SD (y.o.)**	10.8 ± 3.3
**Complaints (No.)**	
Incidentally finding	6 (54.5%)
Headache	4 (36.4%)
Seizure	1 (9.1%)
**Cyst diameter (cm)**	9.7 ± 3.7
**Cyst volume (cm**^**3**^)	263.1 ± 202.2
**BPD (cm)**	149.4 ± 11.1
**OFD (cm)**	177.8 ± 10.1

Abbreviations: No. Number of cases; y.o. year-old; BPD biparietal diameter; OFD occipitofrontal diameter

### Cognitive results in the WISC-IV and CNS VS

**Fig. 1:**
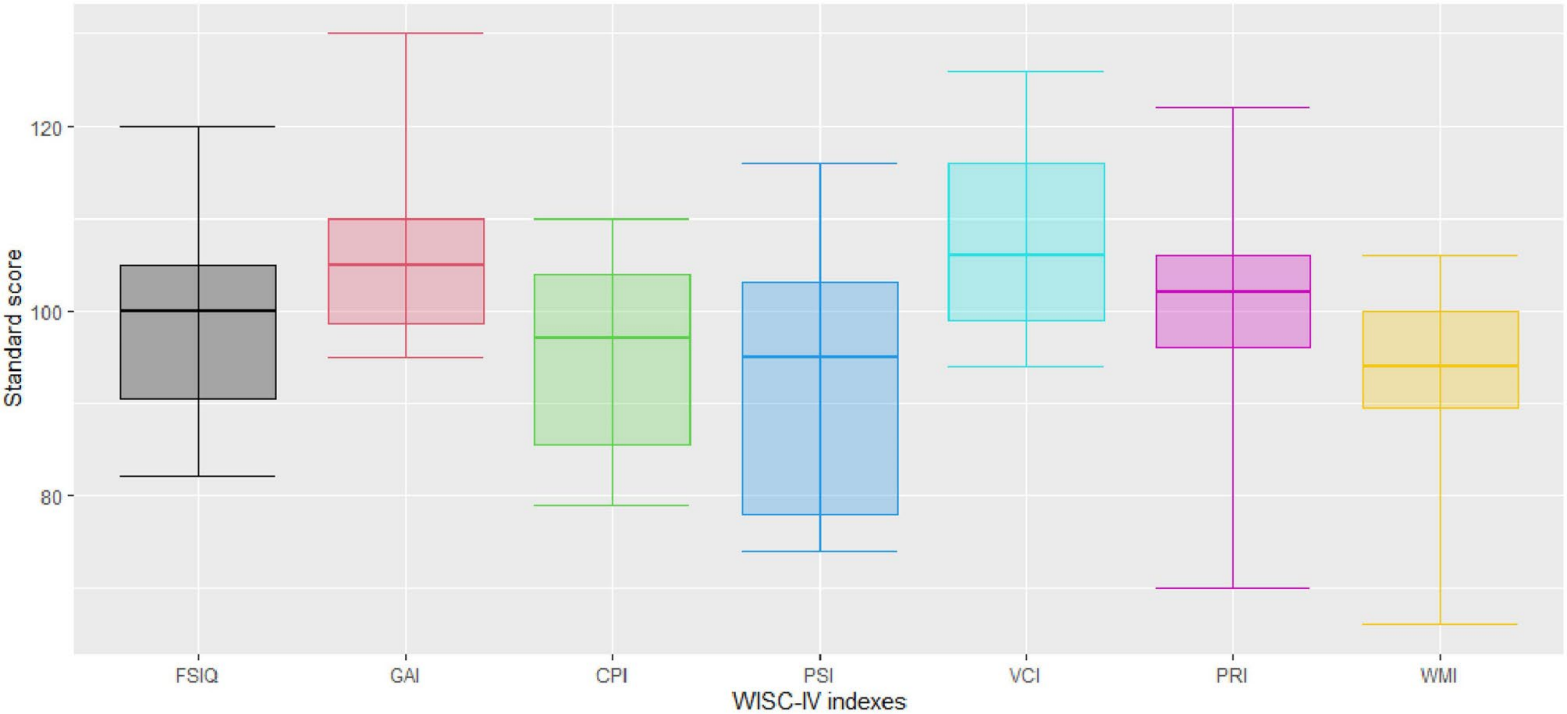
Standard scores of the WISC-IV battery in MFAC patients. Boxes represent performance ranging from the 25th percentile (bottom of box) to the 75th percentile (top of box) of scores. The thin vertical lines represent the minimum (bottom of line) scores to the maximum (top of line) scores obtained. The middle lines represent the median scores. Abbreviations were shown in Supplementary material 2. Refer to Supplementary material 1 for the raw data of the patient group

In the WISC-IV, the performance of MFAC patients was within ± 1 Z-score of the normative level (Fig. 1).

**Fig. 2:**
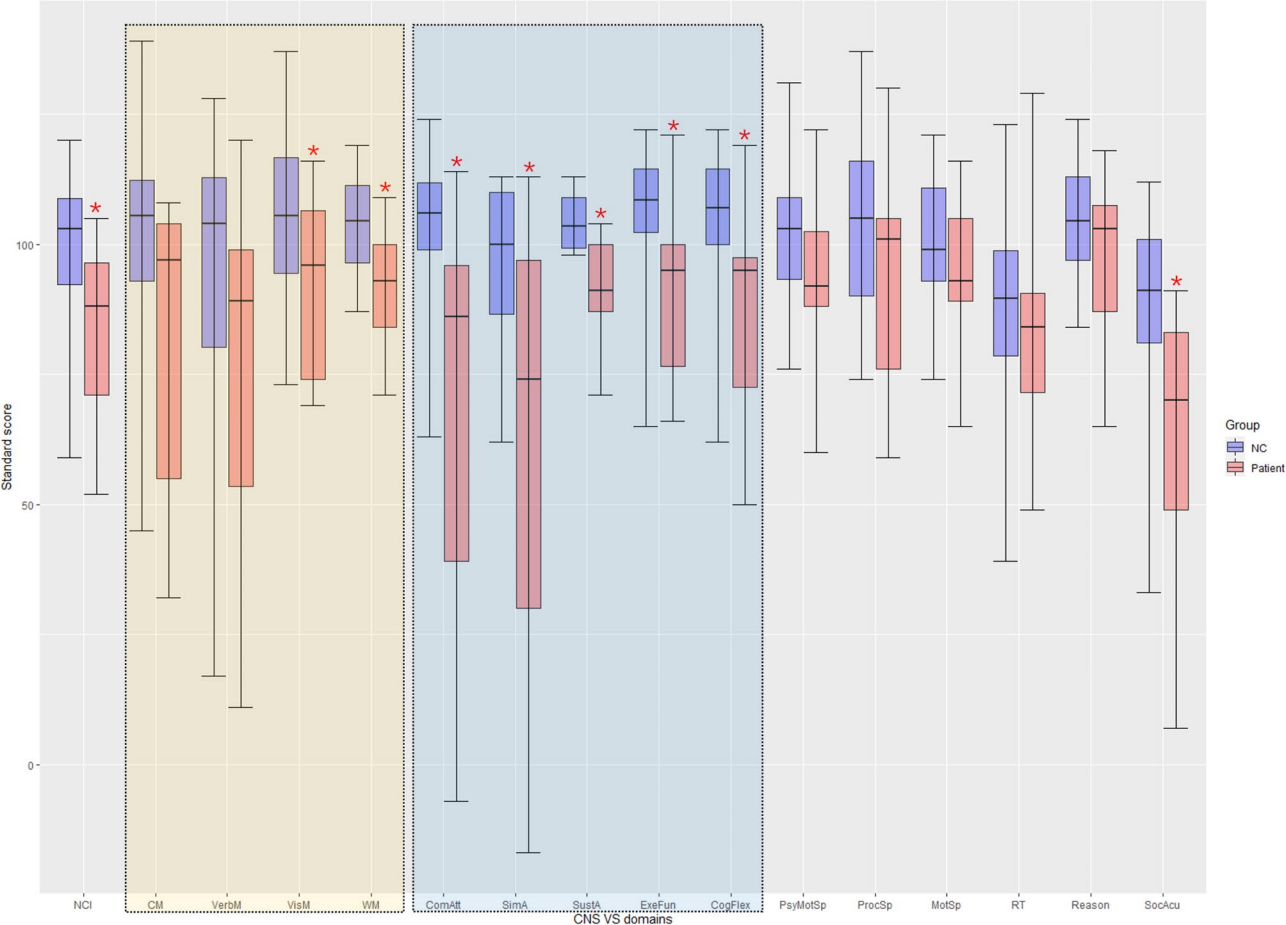
The standard score of CNS VS battery in MFAC patients. The boxes represent performance ranging from the 25th percentile (bottom of box) to the 75th percentile (top of box) of scores. The thin vertical lines represent the minimum (bottom of line) scores to the maximum (top of line) scores obtained. The middle lines represent the median scores. The yellow box indicates the memory-related domains. The blue box indicates the attention-related domains. The “*” indicates a significant statistical difference (*p-unc* < 0.05). Abbreviations were shown in Supplementary material 2. Refer to Supplementary material 1 for the raw data of the patient group

In the CNS VS, patients showed inferior performance in all domains, where 60% (9/15) of domains in patients were significantly lower than the NC group, including NCI (patients vs. NC: 82.8 ± 18.7 vs. 100.1 ± 13.4, *t* = 3.052, *p-unc* = 0.005), Visual Memory (92.8 ± 17.9 vs. 106.7 ± 16.2, *t* = 2.239, *p-unc* = 0.032), Working Memory (91.4 ± 10.7 vs. 103.6 ± 9.1, *t* = 3.438, *p-unc* = 0.002), Complex Attention (68.6 ± 38.5 vs. 103.9 ± 12.8, *t* = 2.956, *p-unc* = 0.013), Simple Attention (61.9 ± 45.6 vs. 96.1 ± 16.1, *t* = 2.417, *p-unc* = 0.034), Sustained Attention (90.8 ± 10.8 vs. 104.2 ± 5.2, *t* = 3.906, *p-unc* = 0.002), Executive Function (91.3 ± 18.9 vs. 105.2 ± 14.4, *t* = 2.364, *p-unc* = 0.025), Cognitive Flexibility (86.0 ± 20.1 vs. 104.4 ± 14.8, *t* = 2.978, *p-unc* = 0.006), and Social Acuity (60.4 ± 29.8 vs. 88.6 ± 17.9, *t* = 2.896, *p-unc* = 0.012) (Fig. 2).

### Functional connectivity in rs-fMRI

**Fig. 3:**
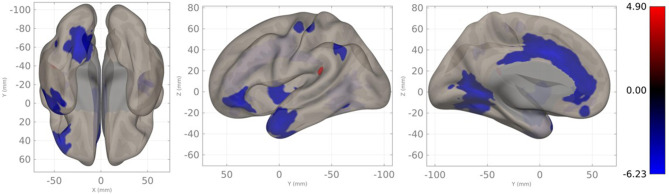
A whole-brain local correlation analysis for the patient group and normal control group (voxel threshold *p-unc* < 0.001, cluster-size threshold of *p-FDR* < 0.05). Red: patient group > normal control group; Blue: patient group < normal control group

A whole-brain LCOR analysis of the patient group and the NC group was conducted. It showed an extensively lower LCOR in the patient group (Fig. 3). The largest area located on the left anterior cingulate cortex (ACC, center MNI [-06 + 14 + 30], voxel size = 1091 voxels, *t* = -7.30, voxel threshold *p-unc* < 0.001, cluster-size threshold of *p-FDR* < 0.001).

**Fig. 4:**
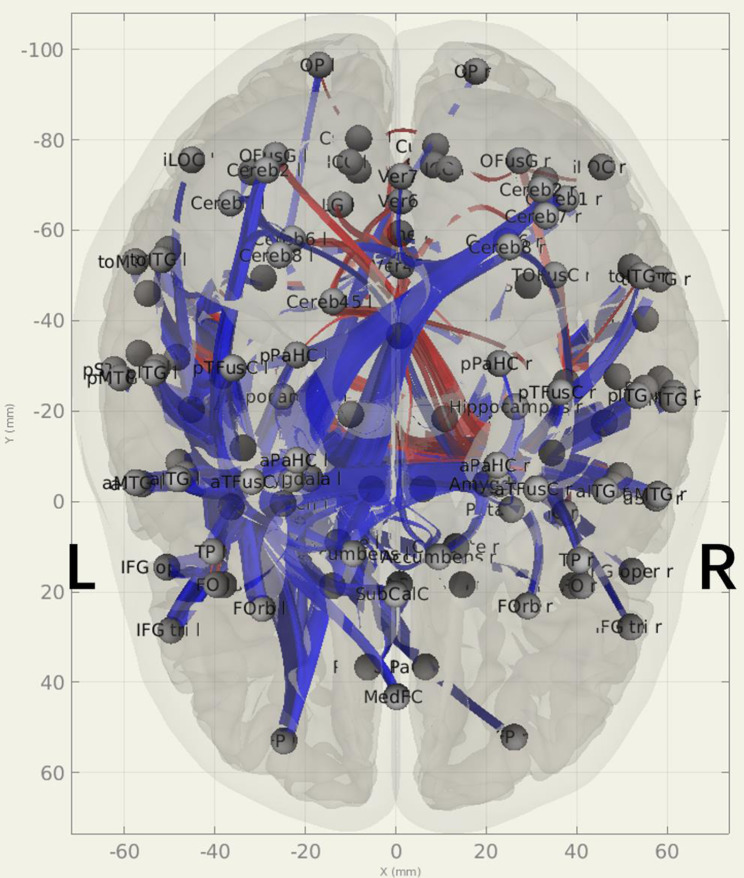
Connections matrix of 132 ROI cortical and subcortical areas for the patient group and normal control group (*p-FDR* < 0.05, only statistically significant connections remained). Red: patient group > normal control group; Blue: patient group < normal control group. The nomenclature of the ROIs were shown in the Supplementary material 3

Figure 4 illustrated the connections among 132 ROI cortical and subcortical areas between the patient group and the NC group (only statistically significant connections remained). It showed that the bilateral frontal and temporal lobes connectivity in the patient group was significantly lower than the NC group (*p-FDR* < 0.05).

**Fig. 5:**
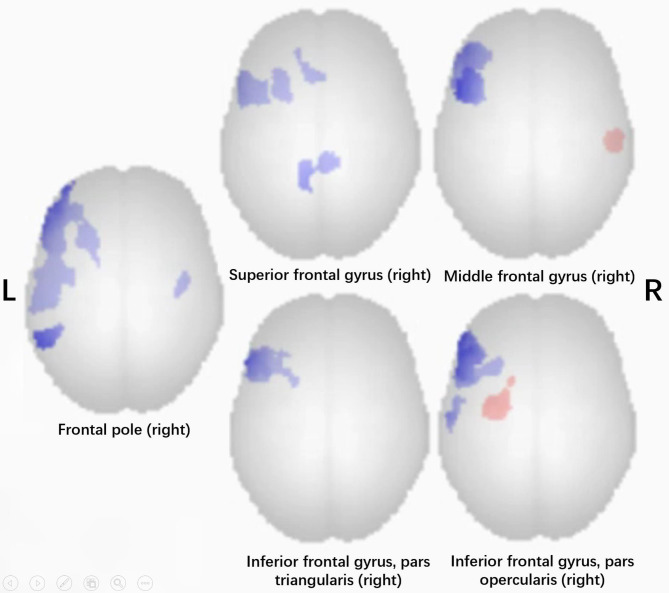
Seed-based FC analysis using five ROIs in the right frontal lobe for the patient group and normal control group (voxel threshold *p-unc* < 0.001, cluster-size threshold of *p-FDR* < 0.05). Blue: patient group < normal control group

**Fig. 6:**
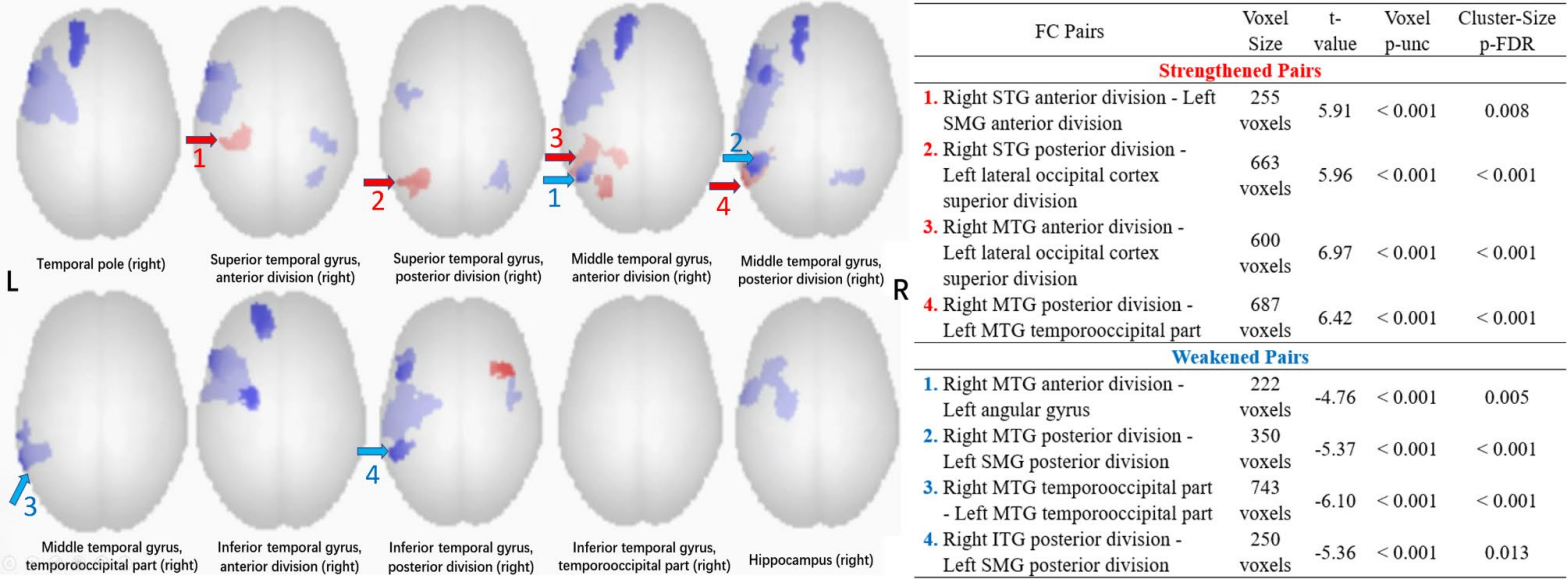
Seed-based FC analysis using 10 ROIs in the right temporal lobe for the patient group and normal control group (voxel threshold *p-unc* < 0.001, cluster-size threshold of *p-FDR* < 0.05). Blue: patient group < normal control group. Arrows indicated FC pairs used in the cognitive data correlation analysis

Seed-based FC analysis was performed to calculate the FC difference in the frontal and temporal lobes between the patient group and the NC group. A total of 15 ROIs in the right hemisphere were chosen as seed points. Figure 5 demonstrated the FC difference for five ROIs in the right frontal lobe. No significant strengthened FC was observed in the left hemisphere (voxel threshold *p-unc* > 0.001, cluster-size threshold of *p-FDR* < 0.05). Figure 6 demonstrated the FC difference for 10 ROIs in the right temporal lobe. It showed that there was significantly altered FC between the right temporal lobe and the left temporooccipital area.

### Correlations of the cognitive assessment

**Fig. 7:**
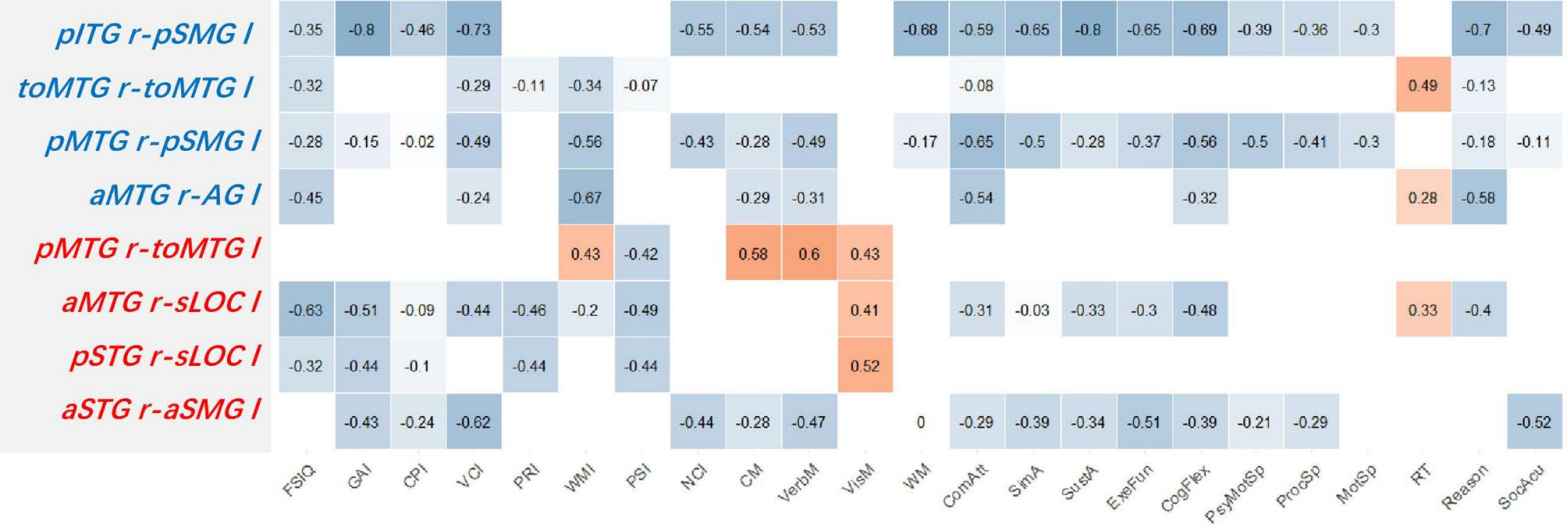
The correlation matrix of the CNS VS, WISC-IV, and Z-transformed score of the selected FC pairs. The Spearman correlation coefficients (r) are shown in the matrix. Non-significant (*p-FDR* > 0.05) coefficients are left blank. The closer the r to the ± 1 indicates the stronger correlation. Red font: FC pairs that patient group > normal control group; Blue font: FC pairs that patient group < normal control group. Abbreviations were shown in Supplementary material 2

The correlations between patients’ cognitive data and the altered FC pairs mentioned above were illustrated in Fig. 7. It showed that the weakened FC pairs (font color blue) negatively correlated to most domains of the WISC-IV and CNS VS. The right ITG posterior division-left SMG posterior division connectivity exhibited the strongest negative effect on WISC-IV (high correlation: VCI) and CNS VS (high correlation: Sustained Attention, and Reasoning). For the strengthened FC pairs, the right MTG posterior division-left MTG temporooccipital part displayed positive correlations to memory-related domains in both WISC-IV (low correlation: WMI) and CNS VS (low-moderate correlation: Composite Memory, Verbal Memory and Visual Memory).

**Fig. 8:**
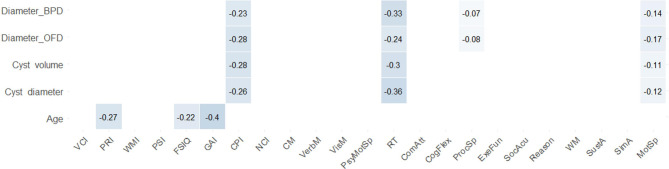
The correlation matrix of the CNS VS, WISC-IV, and clinical variables. The Spearman correlation coefficients (r) are shown in the matrix. Non-significant (*p-FDR* > 0.05) coefficients are left blank. The closer the r to the ± 1 indicates the stronger correlation. Abbreviations were shown in Supplementary material 2

The correlations between patient clinical data and cognitive data were shown in Fig. 8. Five clinical data including age, cyst largest diameter, cyst volume, diameter/BPD and diameter/OFD were included. It showed that most domains in the WISC-IV and CNS VS were not related to the clinical indexes (*p-FDR* > 0.05), except that Reaction Time in the CNS VS showed low correlations to the cyst size (-0.30–-0.36, *p-FDR* < 0.05).

## Discussion

Below we discuss three questions: (1) How were MFAC patients different from healthy children in cognitive function? (2) How did the MFAC affect FC in patients’ rs-fMRI? (3) Did the healthy hemisphere compensate for the affected side in the patients?

### Cognitive impairment in large left MFAC

Frontal and temporal lobes play critical roles in attention and memory [20, 21]. Large MFAC partially or totally occupies frontal and temporal lobes. As we expected, the patient group had a significantly lower attention (Fig. 2, blue box) and memory (Fig. 2, yellow box) ability than the NC group in the CNS VS. However, in the WISC IV, the patient group had standard scores within ± 1 standard deviation, which indicated that MFAC patients were “normal”.

This implied that the ability to detect cognitive changes in MFAC patients highly depended on the battery used. In our study, the CNS VS revealed significantly more neurocognitive deficits than WISC-IV. The WISC-IV scale focused on intellectual functioning, and it revealed that MFAC patients in our study had normal intelligence. This could be attributed to the inadequate sensitivity of WISC-IV in detecting neuropsychological dysfunction [22].

### Altered local and distant FC in MFAC patients

**Fig. 9:**
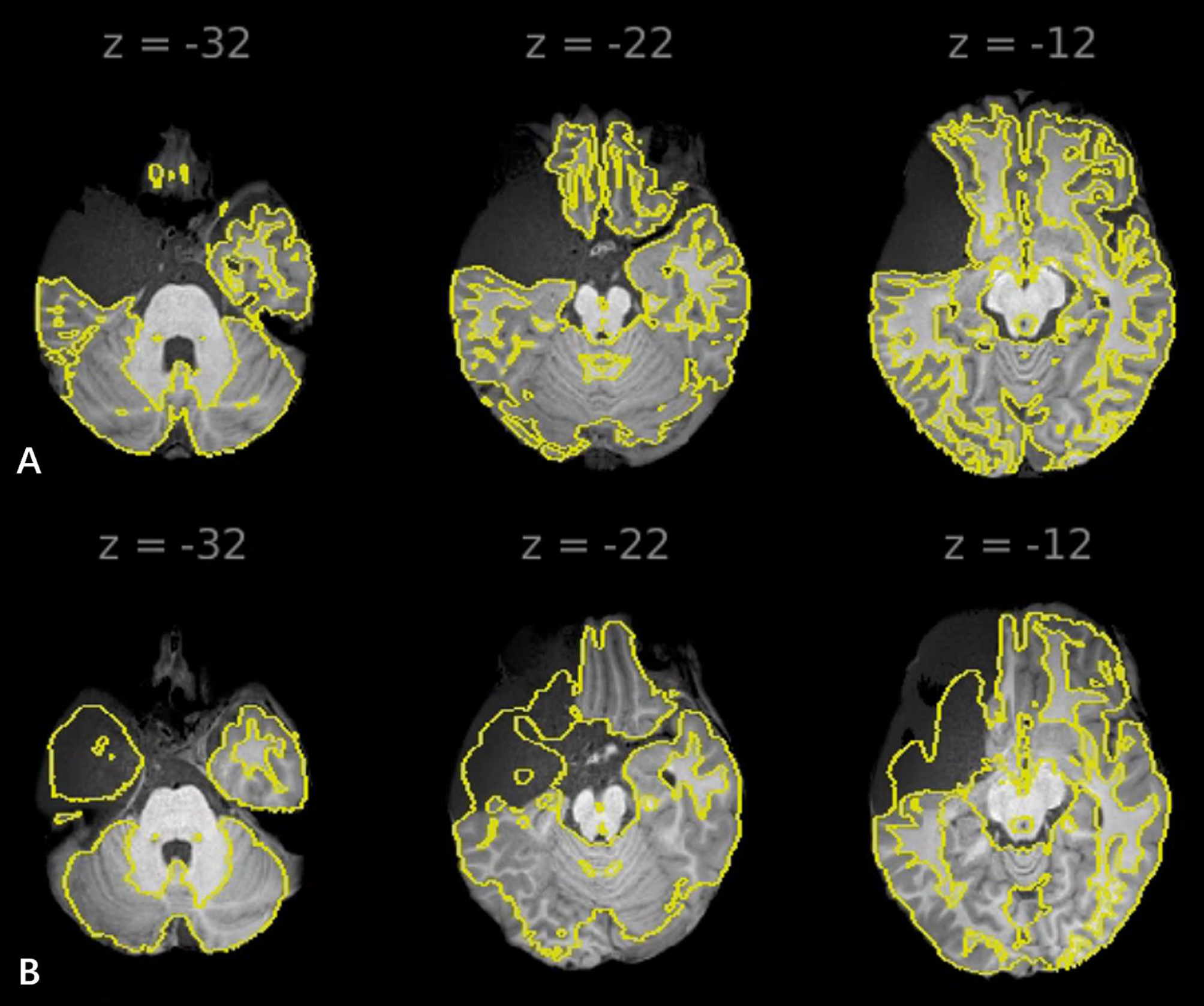
MFAC anatomical data’s co-registration and normalization in the CONN toolbox. (**A**) Well-aligned T1-weight image for a patient with left MFAC ; (**B**) Poor alignment in the left temporal lobe for a patient. It was noticed that the structures of the midline, right hemisphere and left temporal-occipital area remained well aligned

The biggest obstacle in MFAC fMRI study lies in the structural co-registration. In the CONN toolbox, the co-registration and normalization for anatomical data are under iterative tissue classification [23]. As the MRI signal of MFACs varied in terms of the fluid composition, it was unlikely to guarantee an accurate co-registration of the left frontal-temporal ROIs in each patient (Fig. 9). We found that the co-registration of the midline, right hemisphere and left temporal-occipital area was unimpeded. The FC analysis in these areas was still reliable.

Maturation of children brain networks is a trend of decreases in short-range functional connections (assessed by LCOR in this study), and increases in long-range functional connections (assessed by seed-based FC analysis in this study) [24]. LCOR (Fig. 3) indicated that the patient group had generally weakened connectivity than the NC group. The most remarkable change occurred in the left frontal and temporal lobes, which was not surprising due to the loss of BOLD signal in the cyst. There was also a diffuse lower LCOR in the left ACC in the patient group. ACC is a key node of the salience network, which aids in switching between the central executive and default mode networks [25]. Furtherly, ACC activated during pain perception and was positively correlated to the empathy scale [26]. This may be related to the poorer performance of attention domains and Social Acuity in the patient group than the NC group.

Figure 4 indicated that distant FC also weaken in the majority of the ROIs in the patient group. While the weakened FC in the left hemisphere was reasonable, the compensation (strengthened FC) in the right hemisphere was few. A similar AC research utilizing fMRI was published by Rechtman et al. They conducted a voxel-wise whole-brain analysis to investigate rest cerebral blood flow abnormalities in posterior fossa AC. They found significant bilateral decreases in blood flow in these patients compared to controls in both STG and the temporoparietal junction [27]. They concluded that the development of posterior fossa AC early in life may disrupt the maturation of distant temporal regions, by the phenomena of developmental diaschisis [28]. Our result coincided with Rechtman et al. Both weakened local and distant connectivity in the patient group suggested that there could be developmental diaschisis in MFAC children.

### Did neuroplasticity occur in MFAC patients?

If MFAC is a progressive enlarging space-occupying lesion, according to the Grafman theory, neuroplasticity such as “map expansion” (enlargement or displacement of a functional brain region) or “homologous space adaptation” (assumption of a certain cognitive process by a homologous region in the opposite hemisphere) should have occurred [11]. Stowe et al. studied language localization in four adults having left MFAC using positron emission tomography [29]. They found that adult patients did not have “homologous space adaptation” in the right hemisphere.

As we were interested in the neuroplasticity in the opposite hemisphere, we evaluated seed-based FC of the right frontal and temporal lobes. We excluded the significant FC changes located in the MFAC area due to the loss of BOLD signal in the area. After that, we found no evident change of FC in the right frontal lobe (Fig. 5). In the right temporal lobe, the right STG and MTG had strengthened FC in the left temporal-occipital cortex (four pairs, red arrows in Fig. 6). We also noticed that the right MTG and ITG had weakened FC in the left SMG and MTG, around the left temporal-parietal area (four pairs, blue arrows in Fig. 6).

The above altered FC in the frontal-temporal lobe of the patient group was primarily inter-hemispherical connections. Numerous rs-fMRI studies had suggested that inter-hemispherical FC was critical for predicting cognitive deficits and recovery in neurological disease [30, 31]. Bitemporal lobes connections had been thought to support the conceptual representations for both verbal and visual stimuli [32]. Accordingly, the decreased FC pairs in the patient group (four blue rows in Fig. 7) showed negative correlations to most of the cognitive scores. Surprisingly, we found that the strengthened FC pairs between the right temporal lobe and left temporal-occipital/parietal lobe (four red rows in Fig. 7) also negatively correlated to most of the other cognitive domains. Yet, there were still signs of neuroplasticity. We found that the higher connectivity in the right MTG posterior division-left MTG temporooccipital part (Fig. 7, the 1st red row) contributed to the higher Verbal/Visual Memory score in the CNS VS and WMI score in the WISC-IV (low-moderate correlation). This phenomenon best fitted the “map expansion” theory, as it suggested a possible functional compensation of the left MTG temporooccipital part for the disappeared left temporal lobe. The same phenomena were observed in the left lateral occipital cortex superior division (Fig. 7, the 2nd and 3rd red row), where the inter-hemispherical FC also positively correlated to Visual Memory (low-moderate correlation).

The FC pattern we observed in the patient group was more like a systemic disease, such as autism [33] and depression [34]. These diseases usually presented as a reduced inter-hemispheric FC in large-scale brain networks, which was associated with patients’ poor cognitive or social performance. Our results suggested that the cognitive dysfunction in MFAC patients may not be simply attributed to the mass effect of the cyst, but rather a result of whole-brain connectivity aberration.

### Correlations between clinical data and cognition

Kwiatkowska et al. studied the effect of age and cyst size on cognitive deficits in 32 children with MFAC aged between 5 and 17 years old [35]. Their results indicated that cognitive deficits became more pronounced with age, and cyst size negatively correlated with children’s reasoning abilities. In contrast to Kwiatkowska, we found that age and cyst size were not significantly associated with most domains in either WISC-IV or CNS VS batteries (Fig. 8). The primary reason could be the different patient inclusion criteria. Kwiatkowska’s study included various types of MFAC, while in our study, only type III MFAC was included. Large MFAC may already cause a dramatic cognitive dysfunction, hence the weight of cyst size became less statistically important.

To adjust the confounding effect of skull size, we also calculated the ratio between MFAC diameter and the skull measurement parameters (BPD and OFD). Doing so yielded a similar result.

## Conclusions

To our knowledge, this is the first rs-fMRI study in MFAC patients. We concluded the following in school-aged children with large left MFAC: (1) Patients showed significantly impaired cognitive function, primarily in attention and memory domains; (2) There was extensively weakened whole-brain functional connectivity in the patients. Neuroplasticity in the opposite hemisphere was sparse. (3) Cognitive performance was irrelevant to patients’ age and MFAC size.

### Limitations

The major limitation of our study was that the sample size was relatively small. Could neuroplasticity play a more prominent role in MFACs with smaller sizes (type I and II)? Including more patients can better delineate the picture.

### Electronic supplementary material

Below is the link to the electronic supplementary material.

Supplementary Material 1

Supplementary Material 2

Supplementary Material 3

Supplementary Material 4

## Data Availability

The cognitive data can be obtained from the supplementary material 1.
