# Supplementary material for: Does right hemisphere compensate for the left in school-age children with large left middle fossa arachnoid cysts?
Source: BMC Pediatr. 2023 Nov 3;23:550. doi: 10.1186/s12887-023-04148-1 (PMC10623878; doi:10.1186/s12887-023-04148-1)
Supplement: Supplementary file 2 — Supplementary Material 2 [file 12887_2023_4148_MOESM2_ESM.docx]

**Appendix 1. List of abbreviations**

ACC: Anterior Cingulate Cortex

ACs: Arachnoid Cysts

AG: Angular Gyrus

BPD: Biparietal Diameter

CNS VS: CNS Vital Signs

CompM: Composite Memory

ComplAtt: Complex Attention

CogFlex: Cognitive Flexibility

CPI: Cognitive Proficiency Index

Diameter_BPD: diameter/biparietal diameter

Diameter_OFD: diameter/occipitofrontal diameter

ExeFun: Executive Function

FC: Functional Connectivity

FOV: Field of View

FSIQ: Full-Scale Intelligence Quotient

GAI: General Ability Index

IFG tri: Inferior Frontal Gyrus pars triangularis

ITG: Inferior Temporal Gyrus

LCOR: Local Correlation

l: Left

MFACs: Middle Fossa Arachnoid Cysts

MNI: Montreal Neurological Institute

MotSp: Motor Speed

MTG: Middle Temporal Gyrus

NC: Normal Control group

NCI: Neurocognition Index

NC: Normal Controls

OFD: Occipitofrontal Diameter

PRI: Perceptual Reasoning Index

ProcSp: Processing Speed

PsyMotSp: Psychomotor Speed

p-FDR: False Discovery Rate adjusted p-value

p-unc: Uncorrected p-value

ROI: Region of Interest

RT: Reaction Time

r: Right

SMS: Simultaneous Multislice

SMG: Supramarginal Gyrus

SocAcu: Social Acuity

SimpAtt: Simple Attention

SustAtt: Sustained Attention

STG: Superior Temporal Gyrus

VC: Verbal Comprehension Index

VisM: Visual Memory

WM: Working Memory

WMI: Working Memory Index

WISC-IV: Wechsler Intelligence Scale for Children, Fourth Edition
