# Supplementary material for: Does right hemisphere compensate for the left in school-age children with large left middle fossa arachnoid cysts?
Source: BMC Pediatr. 2023 Nov 3;23:550. doi: 10.1186/s12887-023-04148-1 (PMC10623878; doi:10.1186/s12887-023-04148-1)
Supplement: Supplementary file 4 — Supplementary Material 4 [file 12887_2023_4148_MOESM4_ESM.docx]

Supplementary material 1. The standard score of domains in the WISC-IV and the CNS VS of 11 patients (Supplementary 1. 11 MFAC.xlsx).

Supplementary material 2. Abbreviations in the article.

Supplementary material 3. The nomenclature of 164 ROIs in CONN toolbox (Supplementary 2. 164 ROIs.xlsx).
